# Ovarian Expression of Adipokines in Polycystic Ovary Syndrome: A Role for Chemerin, Omentin, and Apelin in Follicular Growth Arrest and Ovulatory Dysfunction?

**DOI:** 10.3390/ijms20153778

**Published:** 2019-08-02

**Authors:** Alice Bongrani, Namya Mellouk, Christelle Rame, Marion Cornuau, Fabrice Guérif, Pascal Froment, Joëlle Dupont

**Affiliations:** 1 Institut National de la Recherche Agronomique Unité Mixte de Recherche Physiology Department, Physiologie de la Reproduction et des Comportements, F-37380 Nouzilly, France; 2 Centre National de la Recherche Scientifique, Life Science Department Physiologie de la Reproduction et des Comportements, F-37380 Nouzilly, France; 3 Université François Rabelais de Tours, F-37041 Tours, France; 4 Institut Français du Cheval et de l’équitation F-37380 Nouzilly, France; 5 Service de Médecine et Biologie de la Reproduction, CHRU Bretonneau, 2, boulevard Tonnellé, F-37044 Tours, France

**Keywords:** adipokines, PCOS, polycystic ovary morphology, follicular fluid, human granulosa cells

## Abstract

Adipokines are a potential link between reproduction and energy metabolism and could partly explain some infertilities related to some pathophysiology, such as polycystic ovary syndrome (PCOS). However, adipokines were predominantly assessed in blood samples, while very little is known concerning their variations in follicular fluid (FF) and ovarian granulosa cells (GCs) of PCOS women. Thus, the objectives of our study were to investigate adiponectin, chemerin, resistin, visfatin, omentin, and apelin ovarian expression in PCOS women in comparison with controls and women with only a polycystic ovary morphology. In total, 78 women undergoing an in vitro fertilization procedure were divided into three groups: 23 PCOS women, 28 women presenting only ≥12 follicles per ovary (ECHO group), and 27 control women. Each group almost equally included normal weight and obese women. Follicular fluid (FF) concentration and granulosa cells (GCs) mRNA expression of adipokines and their receptors were assessed by ELISA and RT-qPCR, respectively. Omentin levels in FF and GC were higher in PCOS than in ECHO and control women, while apelin expression was increased in both PCOS and ECHO groups. FF chemerin concentration was predominant in normal-weight PCOS women compared to BMI (Body Mass Index)-matched ECHO and control women, while GC mRNA levels were higher in the obese PCOS group than in the ECHO one. Compared to PCOS, ECHO women had increased FF adiponectin concentrations and lower plasma AMH levels. The FF concentration of all adipokines was higher in obese subjects except for adiponectin, predominant in normal-weight women. In conclusion, women with PCOS expressed higher GC chemerin and omentin, whereas the ECHO group presented higher levels of FF adiponectin and apelin and lower plasma AMH and LH concentrations. Chemerin, omentin, and apelin expression was differently regulated in women with PCOS, suggesting their possible role in follicular growth arrest and ovulatory dysfunction characterizing PCOS pathogenesis.

## 1. Introduction 

Polycystic ovary syndrome (PCOS) is a very common endocrinopathy affecting 6% to 13% of women of reproductive age and is one of the leading causes of female poor fertility [1]. It was initially described as the association of anovulation and clinical and/or biological hyperandrogenism (1990 National Institutes of Health-Sponsored conference). In 2003, the Rotterdam Consensus Conference introduced polycystic ovaries on ultrasound (corresponding to a follicle number per ovary ≥12 and/or an ovarian volume ≥10 mL) as a supplementary, not mandatory, diagnostic criterion [2]. Thus, PCOS diagnosis currently requires the presence of at least two of these three criteria: Oligo/anovulation, hyperandrogenism, and polycystic ovaries morphology (PCOM) [3]. Despite its typical association with insulin resistance (IR), abdominal obesity [4], and an increased risk of developing type 2 diabetes [5], the causal relationship between reproductive and metabolic features in PCOS has not yet been fully elucidated. Adverse effects of obesity on fertility have largely been discussed and investigated [6,7]. Notably, in PCOS, it has been suggested that an original adipose tissue dysfunction, possibly due to in utero androgen hyperexposition and leading to excessive visceral fat depots, may play a key role in determining both IR and altered androgen metabolism [8]. 

It is well known that white adipose tissue can act as a metabolically active tissue able to synthetize and secrete many endocrine compounds called adipokines [9,10]. The involvement of these molecules in human fertility has been earning growing interest in the last years. Leptin implication in the interaction between energy metabolism and the reproductive system is nowadays widely admitted [11,12]. More recently, it has been demonstrated that several other adipokines may play a role in female reproductive function, and notably in ovarian physiology [13]. Indeed, in vitro investigations demonstrated that adiponectin receptors AdipoR1 and AdipoR2 [14], chemerin and its receptor chemerin chemokine-Like Receptor 1 (CMKLR1) [15], resistin [16], visfatin [17], omentin [18], apelin, and its receptor APJ (Apelin Receptor) [19] are expressed at both mRNA and protein levels in human granulosa cells (GCs). Moreover, some of these adipokines have been in vitro demonstrated to be implicated in human ovarian follicle function [20] and modulation of steroidogenesis [14,15,16,17,19,21]. Plasma levels of adiponectin [4], chemerin [22,23], resistin [24,25], visfatin [26,27], omentin [28,29], and apelin [19] seem to vary in women with PCOS, but the literature is poor, and results are often discordant [19,30,31,32,33,34]. Further, adipokines were predominantly assessed in blood samples, while very little is known concerning their variations in follicular fluid (FF) and GCs of women with PCOS [19,35,36,37].

The aim of our study was to investigate the concentration in FF and the mRNA expression in GC of adiponectin, chemerin, resistin, visfatin, omentin, and apelin and some of their receptors in PCOS women. Further, to improve the understanding of PCOS etiology that is still under debate, and to identify adipokines potentially involved in its physiopathology, we chose to compare PCOS subjects to a cohort of women presenting only PCOM on ultrasound, without any other characteristic feature of PCOS. This condition, which we named “ECHO”, has in addition significant clinical interest, since it is well known that a high number of ovarian follicles is a major risk factor of ovarian hyperstimulation syndrome during a medically assisted reproduction (MAR) procedure [38].

## 2. Results 

### 2.1. Anthropometric, Clinical, and Hormonal Data

**Figure 1:**
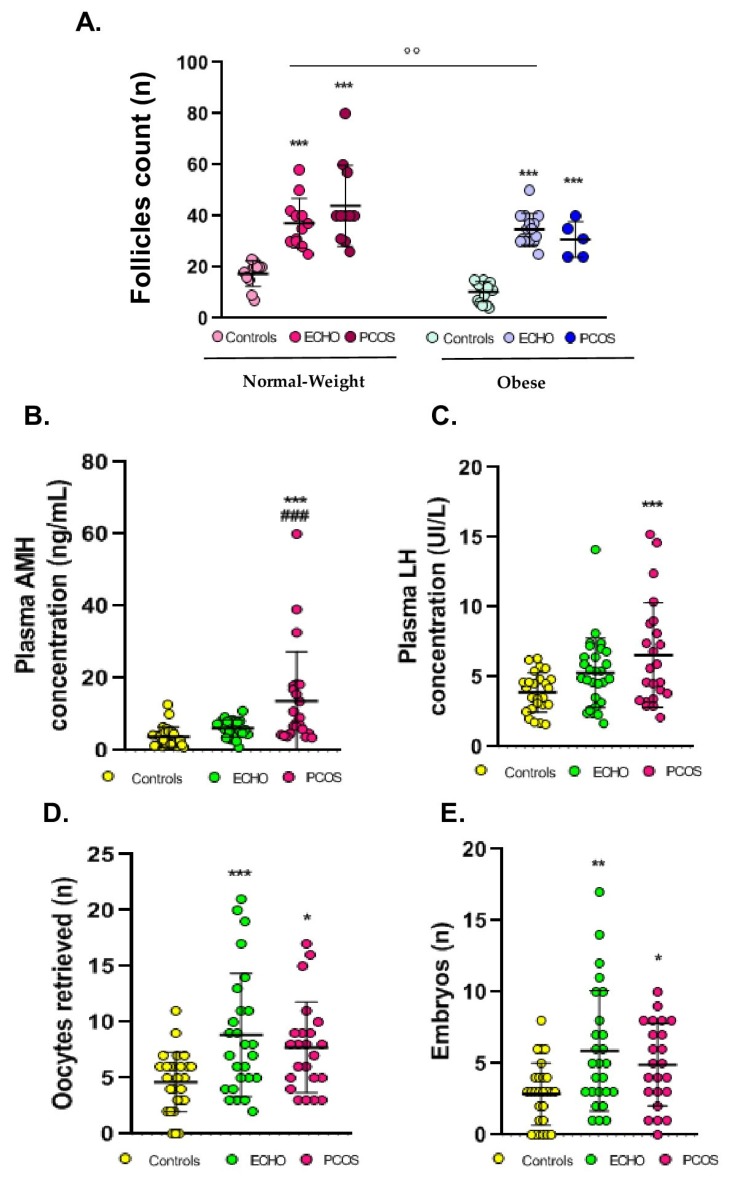
Clinical parameters, hormonal data, and in vitro fertilization procedure outcomes in patients of different studied groups. (**A**) Follicle counts assessed by transvaginal ultrasound, (**B**) plasma AMH concentration, (**C**) plasma LH concentration, (**D**) number of oocytes withdrawn during transvaginal retrieval, and (**E**) number of embryos obtained after in vitro fertilization. For A panel, see Table 1 for the data number per group. For the **B**, **C**, **D**, and **E** panels, the data were pooled in three groups (*n* = 27, *n* = 28, *n* = 23 for controls, ECHO, and PCOS groups, respectively) according to the pathological condition, as the BMI effect was not significant and no interaction between the BMI and pathological condition effect was found. The values are expressed as mean ± standard errors of means. °° indicates significant difference (*p* < 0.01) between normal-weight and obese subjects; * indicates significant difference vs. controls (* *p* < 0.05, ** *p* < 0.01, *** *p* < 0.001); ### indicates significant difference (*p*<0.001) vs. ECHO women.

Anthropometric, clinical, and hormonal data as well as IVF procedure outcomes are detailed in Table 1 and Figure 1. As expected, BMI was higher in obese groups and cycles were longer in PCOS compared to ECHO and control women. ECHO and PCOS groups were characterized by a greater follicle count compared to controls; a significant, not predictable difference was also found between normal-weight and obese women. Plasma AMH and LH levels were higher in PCOS women compared to controls. Interestingly, as regards AMH, we observed a highly statistically significant difference also between PCOS and ECHO groups. Concerning IVF procedure outcomes, oocytes and embryos numbers were greater in ECHO and PCOS women. No difference in plasma FSH and oestradiol levels was observed between the groups. No woman of the ECHO and control groups and only 6 out of 23 women with PCOS presented a clinical and/or biological hyperandrogenism. Plasma testosterone concentrations determined with the same kit for each group are reported in Table 1. 

### 2.2. FF Adiponectin and AdipoR1 Expression in GC Varied Mainly According to BMI

**Figure 2:**
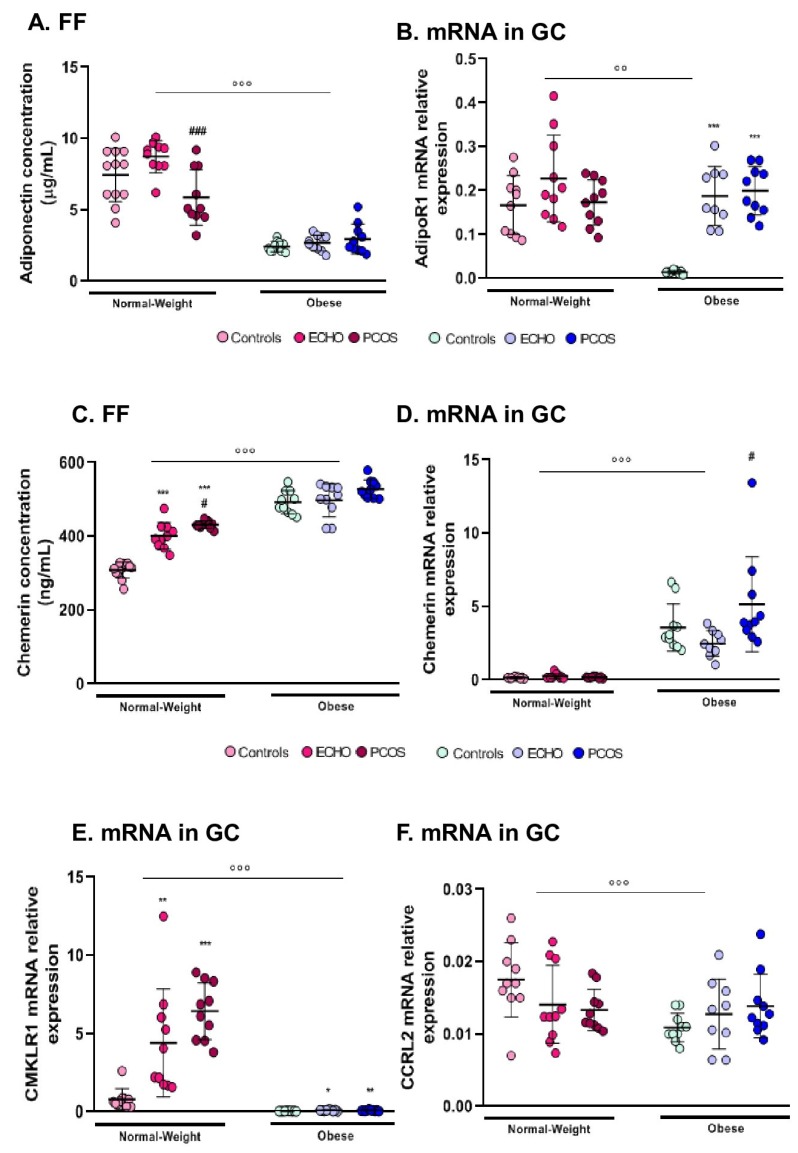
Follicular fluid adiponectin and chemerin concentration and mRNA expression of AdipoR1(Adiponectin receptor 1), CMKLR1, and CCRL2 in granulosa cells of obese and normal-weight PCOS, ECHO, and control groups. (**A**) Follicular fluid (FF) adiponectin concentration assessed by ELISA and (**B**) AdipoR1 mRNA levels in granulosa cells (GCs) quantified by RT-PCR within the six different groups; (**C, D**) chemerin levels in FF and GC; (**E, F**) mRNA levels of CMKLR1 and CCLR2 in GC. The values are expressed as mean ± standard errors of means (*n* = 12 for normal-weight controls, *n* = 13 for normal-weight ECHO, *n* = 13 for normal-weight PCOS, *n* = 15 for obese controls, *n* = 15 for obese ECHO, and *n* = 10 for obese PCOS). ° indicates significant difference between normal-weight and obese subjects (°° *p* < 0.01, °°° *p* < 0.001); * indicates significant difference vs. controls (*** *p* < 0.001); # indicates significant difference vs. ECHO women (# *p* < 0.05, # # # *p* < 0.001).

Adiponectin concentration in FF was clearly significantly higher in normal-weight women then in obese ones (Figure 2A). According to pathological status, we observed a significant difference only within the normal-weight group, with greater levels of adiponectin in ECHO as compared to PCOS women (Figure 2A). Likewise, AdipoR1 was predominantly expressed in GCs of normal-weight women (Figure 2B). However, differences in pathological condition were limited to the obese group, with ECHO and PCOS women showing greater AdipoR1 expression compared to controls (Figure 2B). Both adiponectin concentration in FF and AdipoR1 expression in GCs were negatively correlated with BMI (r = −0.748 and r = −0.288, respectively, Table 2). FF adiponectin was also negatively correlated with plasma E2 (r = −0.30, Table 2), while a positive correlation was observed between AdipoR1 expression and follicles (r = 0.554, *p* < 0.001), oocytes (r = 0.286, *p* < 0.05), and embryos number (r = 0.309, *p* < 0.05). No significant correlation was found between adiponectin concentration in FF and AdipoR1 expression.

### 2.3. Chemerin (FF and GC mRNA Expression) Was Higher in Obese Subjects and in Women with PCOS

Chemerin expression both in FF and GC was greater in obese women than in normal-weight women (Figure 2C,D). Concerning pathological status, chemerin concentration in FF was clearly predominant in women with PCOS, but a statistically significant difference was found only in the normal-weight group (Figure 2C). Otherwise, chemerin expression in GC varied only within the obese group, with significantly higher levels in PCOS compared to ECHO women (Figure 2D). Interestingly, chemerin follicular concentration positively correlated with chemerin mRNA levels in GC (r = 0.64, *p* < 0.001) and both strongly positively correlated with BMI (r = 0.725 and r = 0.694, respectively, Table 2).

### 2.4. CMKLR1 and CCRL2 mRNA Expression in GC was Markedly Reduced in Obese Women

Contrary to chemerin, mRNA levels of its receptor, CMKLR1, were almost undetectable in obese women (Figure 2E). However, in line with what was seen for chemerin, in the normal-weight group, CMKLR1 expression was predominant in women with PCOS, even if the difference with the ECHO group failed to reach statistical significance (Figure 2E). C-C Chemokine Receptor-Like 2 (CCRL2) expression varied only according to BMI, with higher mRNA levels in GCs of normal-weight women compared to the obese ones (Figure 2F). No significant modification was observed according to pathological status. G Protein-Coupled Receptor 1 (GPR1) expression did not change in any condition (data not shown). A positive correlation was observed between CMKLR1 expression and follicle count (r = 0.520, *p* < 0.001), cycle duration (r = 0.337, *p* = 0.01), plasma AMH (r = 0.353, *p* < 0.01), plasma LH (r = 0.306, *p* < 0.05), and plasma E2 concentrations (r = 0.091, *p* < 0.05). Remarkably, CMKLR1 mRNA levels were negatively correlated with BMI (r = −0.622, *p* <.001). Indeed, chemerin expression both in FF and GC was negatively correlated with the mRNA levels of all its receptors, although statistical significance was found only between FF chemerin and CCRL2 (r = 0.342, *p* < 0.05) and between chemerin mRNA levels in GC and CMKLR1 (r = −0.46, *p* < 0.001).

### 2.5. FF Resistin Was Higher in Obese Women

**Figure 3:**
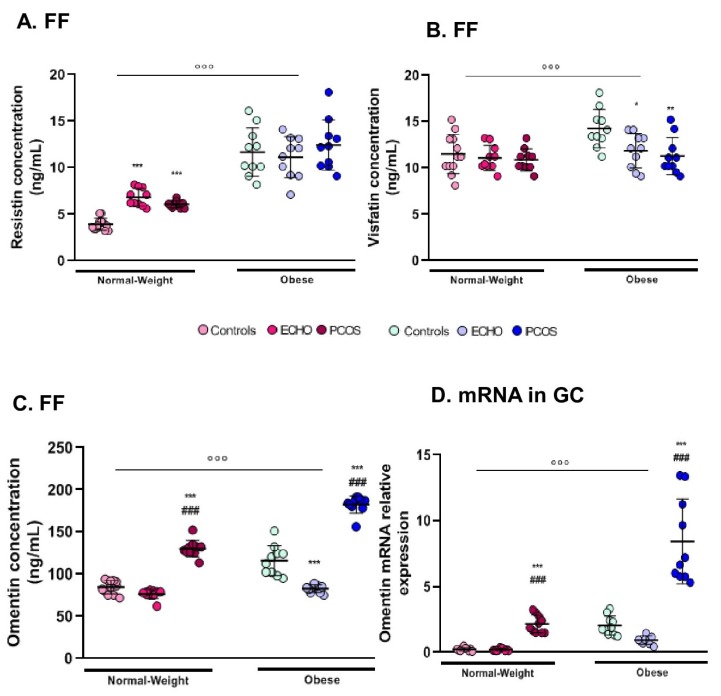
Follicular fluid resistin, visfatin, and omentin concentration and mRNA expression of omentin in granulosa cells of obese and normal-weight PCOS, ECHO, and control groups. **(A)** Follicular fluid (FF) resistin, (**B**) visfatin, and (**C**) omentin concentrations assessed by ELISA; (**D**) mRNA omentin levels in granulosa cells (GCs) quantified by RT-PCR within the six different groups. The values are expressed as mean ± standard errors of means (*n* = 12 for normal-weight controls, *n* = 13 for normal-weight ECHO, *n* = 13 for normal-weight PCOS, *n* = 15 for obese controls, *n* = 15 for obese ECHO, and *n* = 10 for obese PCOS). °°° indicates significant difference (*p* < 0.001) between normal-weight and obese subjects; * indicates significant difference vs. controls (* *p* < 0.05, ** *p* < 0.01, *** *p* < 0.001); ### indicates significant difference (*p* < 0.001) vs. ECHO women.

Resistin concentration in FF was markedly higher in obese women than in the normal-weight ones (Figure 3A) and, interestingly, it was positively correlated with BMI (r = 0.799, Table 2). In the normal-weight group, the highest resistin levels were observed in ECHO and PCOS women (Figure 3A). Resistin mRNA levels in GCs did not vary according to either BMI or pathological condition. Unlike chemerin, FF resistin did not significantly correlate with resistin expression in GCs.

### 2.6. Visfatin Modifications Were Restrained to Its Concentration in FF

As for resistin, visfatin expression varied only in FF and mainly according to BMI, with higher levels in obese subjects compared to the normal-weight ones (Figure 3B). However, follicular concentration of visfatin was lower in ECHO and PCOS women compared to controls, especially in the obese group (Figure 3B). FF visfatin was positively correlated with BMI (r = 0.275, Table 2) and negatively correlated with plasma AMH concentration (r = −0.284) and follicles (r = −0.352), oocytes (r = −0.37), and embryo number (r = −0.262) (Table 2). No significant modification nor correlation was found for visfatin mRNA levels in GCs.

### 2.7. Omentin Expression (FF and GC mRNA) Was Markedly Predominant in Women with PCOS

Omentin concentration was significantly higher in the FF of obese women (Figure 3C). Remarkably, independently from BMI, omentin levels were markedly more elevated in PCOS than in ECHO and control women (Figure 3C). Interestingly, obese ECHO women showed lower omentin follicular concentrations than controls (Figure 3C). The same significant results were found concerning omentin expression in GCs (Figure 3D). Further, omentin concentration in FF was strongly positively correlated with omentin mRNA levels in GCs (r = 0.824, *p* < 0.001) and both positively correlated with BMI (r = 0.446 for follicular omentin, Table 2, and r = 0.464 for GC omentin mRNA levels, *p* < 0.001). A positive correlation was also observed between follicular omentin concentration and cycle duration (r = 0.421, Table 2).

### 2.8. Apelin and Its Receptor APJ Were Mostly Expressed in Obese Subjects and in ECHO/PCOS Women

**Figure 4:**
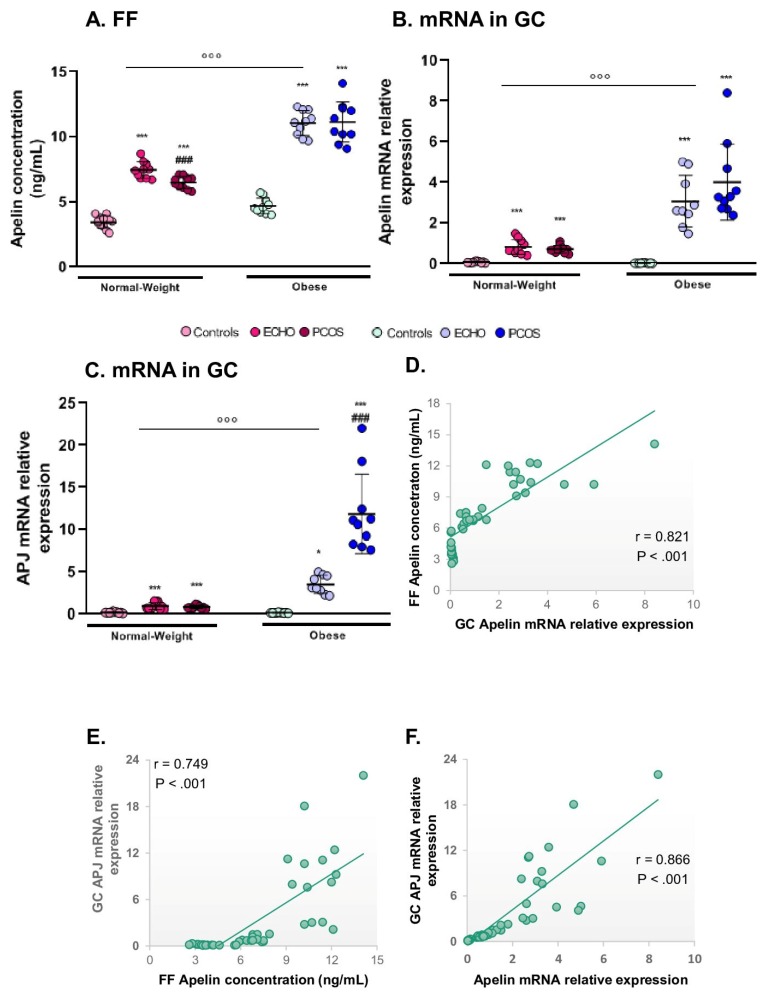
Follicular fluid apelin concentration, mRNA expression of apelin and its receptor, APJ, in GCs of obese and normal-weight PCOS, ECHO, and controls groups and correlations of apelin FF levels and apelin or APJ mRNA expression in GCs. (**A**) Follicular fluid (FF) apelin concentration assessed by ELISA; (**B**) apelin and (**C**) APJ mRNA levels in granulosa cells (GC) quantified by RT-PCR within the six different groups; (**D**) correlation between FF concentration and GC expression of apelin; correlations between APJ mRNA levels in GCs; and (**E**) apelin FF concentration and (**F**) apelin mRNA levels in GCs. The values are expressed as mean ± standard errors of means (*n* = 12 for normal-weight controls, *n* = 13 for normal-weight ECHO, *n* = 13 for normal-weight PCOS, *n* = 15 for obese controls, *n* = 15 for obese ECHO, and *n* = 10 for obese PCOS). °°° indicates significant difference (*p* < 0.001) between normal-weight and obese subjects; *** indicates significant difference (*p* < 0.001) vs. controls; ### indicates significant difference (*p* < 0.001) vs. ECHO women.

Both apelin FF levels and apelin expression in GCs were significantly higher in obese than in normal-weight women (Figure 4A and B) and positively correlated with BMI (r = 0.441, Table 2, and r = 0.554, *p* < 0.001, respectively). According to pathological status, apelin was mostly expressed in ECHO and PCOS women in both the normal-weight and obese groups (Figure 4A and 4B). Interestingly, limited to FF concentration, apelin was significantly lower in normal-weight PCOS than in ECHO women (Figure 4A). A positive correlation was found between follicle count and both apelin levels in FF (r = 0.480, Table 2) and apelin expression in GC (r = 0.301, *p* < 0.05). Follicular apelin also positively correlated with oocytes and embryo numbers (r = 0.30 and r = 0.268, respectively, Table 2) and negatively correlated with plasma E2 concentration (r = −0.284, Table 2). Apelin mRNA levels in GCs were correlated with cycle duration (positive correlation, r = 0.289, *p* < 0.05) and plasma FSH (negative correlation, r = −0.298, *p* < 0.05). Concerning APJ, we found the same significant results, with a predominant expression of this receptor in the obese group and women with PCOS (Figure 4C). Further, like apelin, mRNA levels of APJ positively correlated with BMI (r = 0.510, *p* < 0.001) and cycle duration (r = 0.402, *p* < 0.01) and negatively correlated with plasma FSH concentration (r = −0.282, *p* < 0.05). Notably, APJ expression was strongly positively correlated with apelin expression in GCs (r = 0.866, Figure 4G) and apelin concentration in FF (r = 0.749, Figure 4E right panel), which in turn were strongly positively correlated to each other (r = 0.821, Figure 4D).

## 3. Discussion

Our study aimed to improve the understanding of PCOS etiology, and to identify adipokines potentially involved in its physiopathology. Thus, we analyzed the adipokines’ profile at the ovarian level (FF and GC) in normal-weight and obese women with PCOS diagnosis in comparison with women presenting only a PCOM, a condition that we named “ECHO”. This condition, as discussed above, has per se a significant clinical interest and, to the best of our knowledge, there are no data available about adipokines expression in the FF and GCs of these women. We evaluated adiponectin, chemerin, resistin, visfatin, omentin, and apelin concentrations in FF samples, as well as the expression of the same adipokines and some of their receptors in GCs. The results are discussed below for each single adipokine. 

### 3.1. Adiponectin

Adiponectin is one of the better known and most abundant circulating adipokines. It is mainly produced by white adipocytes and secreted into plasma circulation as three oligomeric complexes, whose medium and high molecular weight isoforms represent 90% of circulating protein. It acts mainly through two G protein-coupled receptors, AdipoR1, which is ubiquitously expressed, and AdipoR2, which is mainly located in white adipose tissue and liver [30]. Its involvement in energy metabolism as an insulin-sensitizing, anti-inflammatory, and anti-atherogenic molecule is largely admitted [30] and obesity and insulin-resistant states have been associated with reduced plasma adiponectin concentrations [4]. In agreement with literature data, we found that adiponectin concentration in FF and AdipoR1 mRNA expression in GCs were markedly higher in normal-weight women then in the obese ones and both negatively correlated with BMI. Interestingly, we also observed that within the normal-weight group, FF adiponectin levels were significantly lower in PCOS compared to ECHO women. Consistent with our findings, despite some conflicting results [30], PCOS women have been reported to present lower adiponectin concentrations in serum [4,39] and FF [35,40], as well as a decreased expression of AdipoR1 and AdipoR2 in adipose tissue [5]. Although adiponectin dysregulation may be one of the possible mechanisms responsible for impairment of insulin-sensitivity in women with PCOS, the reduction of adiponectin levels in serum seems to be independent of IR severity [41]. Thus, adiponectin might play a role in the pathogenesis of other characteristic features of PCOS. In particular, several authors evoked a possible role of this adipokine in folliculogenesis [35,40]. Notably, Campos et al. reported that adiponectin acts directly and indirectly, through the interaction with LH and insulin, on GCs by inducing the expression of genes associated with periovulatory maturation of ovarian follicles [42]. It is therefore noteworthy that in our study, AdipoR1 expression in GCs was positively correlated with follicles, oocytes, and embryo count. On the other hand, contrary to what is reported in the literature [40], we did not find any significant difference in AdipoR2 expression. In addition, mRNA AdipoR1 levels varied only in the obese group, being greater in PCOS and ECHO women compared to controls. The meaning of our findings is currently unknown and deserves to be further elucidated. Although adiponectin involvement in PCOS pathogenesis is supported by several evidences, including genomic analyses [30], it is possible that the limited data we obtained are due to the fact that we only investigated GCs. In our study, we did not find any correlation between FF adiponectin concentration and AdipoR1/AdipoR2 expression in GCs. Hence, since adiponectin levels are twice higher in FF than in plasma [14], theca cells might represent the key actor in the metabolism of adiponectin as the main cell responsible for its production in FF and then the most likely target of its effects.

### 3.2. Resistin

Resistin is a small cysteine-rich protein mainly expressed by macrophages [43] and within adipose tissue, it is predominantly released by omental non-adipocyte resident inflammatory cells [44]. Its relevance and physiological role in humans are currently unclear. Notably, Panidis et al. showed that circulating resistin was higher in overweight women with PCOS but did not differ between PCOS and control women with a normal BMI, although the first were more insulin resistant [45]. Further, after stepwise multiple regression analysis, serum resistin levels were not associated with any parameter independent of BMI, suggesting that they correlated with IR as a consequence of obesity itself, rather than as an independent causative factor [45]. In accordance with these data, in our study, we found that resistin concentration in FF was significantly higher in obese women compared to the normal-weight ones and positively correlated with BMI. As regards PCOS women, some authors noted higher plasma resistin concentrations in overweight/obese patients compared to the normal-weight ones, independently from PCOS diagnosis [45]. However, others showed no difference in serum resistin levels between obese and non-obese PCOS women [25] and most of the studies failed to find a significant correlation between circulating resistin and BMI [37,46]. Interestingly, within the normal-weight group of patients, we observed higher resistin levels in the FF of ECHO and PCOS women compared to controls. Other studies, however, showed no difference in FF resistin concentration between PCOS and healthy normal-weight women [37]. Indeed, the association of resistin with PCOS is largely debated. Resistin mRNA and protein have been detected in granulosa, theca, cumulus cells, and oocytes from human ovarian follicles [16]. In human granulosa cells, recombinant resistin has been reported to decrease IGF-1-induced progesterone and estradiol secretion [16], which was associated with a reduction in P450scc and P450 aromatase levels [16], indicating a role for resistin in the regulation of ovarian steroidogenesis. However, data concerning serum resistin levels in women affected by PCOS are inconsistent. While some authors pointed out significantly higher resistin concentrations in the plasma of women with PCOS [24,25], no difference between PCOS and healthy women was reported by several others [37,45]. Interestingly, resistin mRNA levels in adipocytes were twice higher in women with PCOS compared to controls and significantly decreased after laparoscopic ovarian electrocautery [47,48], suggesting that although systemic resistin does not seem to be actively involved in PCOS pathogenesis, it may act as a local determining factor for this syndrome [45,48]. However, it is noteworthy that in agreement with previous literature data [37], in our study, FF resistin levels did not correlate with any reproductive outcome, making it unlikely that resistin plays a role in oocytes’ maturation and development. Unlike other adipokines, we observed that resistin mRNA levels in GCs did not vary either according to BMI or pathological condition and did not correlate with FF concentration. Resistin levels in FF have been repeatedly found to be lower than in plasma [16,37]. It is therefore unlikely that human GCs, while expressing resistin protein [16], secrete it into FF or circulation. Furthermore, according to our findings and considering that follicular resistin seems to derive primarily from blood plasma, the regulation of resistin expression appears to be different at the systemic and ovarian level.

### 3.3. Visfatin

Visfatin, previously described as a growth factor for early B-cells called pre-B cell colony enhancing factor (PEBF) [49], was later characterized by Fukuhara et al. as a peptide predominantly expressed in and secreted from visceral adipose tissue in both humans and mice [50]. Although visfatin has been proposed as a potential link between visceral obesity and increased metabolic risk [51], data concerning the relationship between this adipokine, obesity, and IR are widely discordant. In our study, visfatin concentration in FF was significantly higher in the obese group than in the normal-weight one and positively correlated with BMI. Despite some conflicting results [52], a recent meta-analysis revealed that plasma visfatin is significantly increased in subjects presenting overweight/obesity, IR, metabolic syndrome, and cardiovascular diseases [53]. A positive association between circulating visfatin and BMI has been reported by several authors [26,27,34,51] but not confirmed by others [52]. On the contrary, the role of visfatin in the regulation of female reproductive functions is supported by several evidences. Indeed, it is expressed in human myometrium, placenta, and human fetal membranes, where it seems to be involved in placentation [17]. In the ovary, its presence has been demonstrated in human follicles, notably in oocytes, cumulus, and GCs and less abundantly in theca cells [17,53]. In human GCs, it has been reported to enhance IGF-1-induced progesterone and estradiol secretion, thus showing a positive effect on steroidogenesis [17]. In our study, we observed significantly lower levels of visfatin in the FF of obese ECHO and PCOS women compared to obese healthy controls, a result not in line with literature data. Indeed, in previous studies, FF visfatin was shown to be similar [54] or higher [36] in women with PCOS when compared to BMI-matched normally ovulatory women. Similarly, as regards circulating visfatin, although two studies failed to highlight a significant difference between PCOS and healthy women [34], most of the authors found significantly higher levels in women with PCOS [26,27,36,54,55]. The decrease in visfatin concentration observed in the FF of the PCOS and ECHO women of our study, which is characterized by a high antral follicle count deriving from follicular growth arrest, suggests a positive effect of visfatin on female reproductive function, and notably folliculogenesis. Indeed, Shen et al. found a significant positive correlation between visfatin concentration in FF and the number of retrieved oocytes [53] and the administration of visfatin during ovulation induction in aged female mice improved the developmental competency of oocytes [56]. However, it needs to be underlined that in our study, follicular visfatin was negatively correlated with follicle, oocyte, and embryo numbers, as well as with plasma AMH concentration. As for resistin, visfatin mRNA levels in GCs did not vary either according to BMI or pathological condition and did not correlate with FF concentration. Visfatin levels in FF have been shown to be similar [53] or lower [54] compared to those in plasma and no correlation was found between visfatin concentrations in plasma and FF [53]. In light of our results, the possibility of an ovarian origin for this adipokine, although previously evoked [53], seems thus unlikely. 

### 3.4. Apelin

Apelin is a bioactive peptide originally identified in bovine stomach extract as the endogenous ligand of the orphan G protein-coupled receptor, APJ [57]. Its expression has been detected in several organs, like the stomach, brain, lung, uterus, and ovary, as well as in the endothelium of small arteries [57], indicating that the apelin/APJ system may play a pivotal role in multiple physiological functions. In particular, apelin seems to be involved in the regulation of food intake, energy metabolism, cardiovascular system, angiogenesis, and neuroendocrine functions [57]. In our study, we found that apelin concentration in FF as well as apelin and APJ mRNA expression in GCs were significantly higher in obese than in normal-weight women and positively correlated with BMI. These results already reported by Roche et al. [19] are consistent also with literature data about circulating apelin levels [58]. As the existence of a correlation between serum apelin levels and IR in PCOS is still a matter of debate [59], apelin, rather than as a marker of insulin sensitivity, may play a role in other characteristic features of PCOS, such as ovulatory dysfunction. Indeed, apelin and APJ expression has been detected in human ovarian follicles, GCs, theca cells, and oocytes and in vitro studies suggest a potential role of apelin in the control of several aspects of ovarian function [19]. Indeed, apelin enhances progesterone and estradiol secretion in human and porcine GC cultures [19,60], it improves rat, bovine, and porcine GC proliferation, and it seems to be involved in the regulation of the bovine corpus luteum luteolysis process [61] and oocyte maturation [62]. In our study, we demonstrated that both apelin concentration in FF and apelin/APJ mRNA expression in GCs positively correlated with antral follicle count and were significantly higher in PCOS and ECHO groups, both characterized by the accumulation of small antral follicles resulting from the failed selection of a dominant follicle. Thus, according to these observations, apelin may play a key role in follicular growth arrest at the origin of PCOM. In support of this hypothesis, apelin has been suggested to be implicated in bovine follicular atresia [63] and in different animal species; both protein and mRNA levels of apelin and APJ have been reported to change during follicular growth, with the highest expression in large follicles [60,63]. Folliculogenesis disruption in PCOS is thought to be due to an increased responsiveness to FSH in terms of oestradiol and progesterone production and to a premature responsiveness to LH in small follicles [64]. Consequently, in PCOS anovulatory women, plasma estradiol levels are slightly higher and FSH levels are lower than in the normal early follicular phase [64]. While keeping in mind that correlations may be merely spurious, without causative significance, very interestingly, we found that apelin mRNA levels in GCs were negatively correlated with plasma FSH levels and positively correlated with cycle duration, strongly supporting that apelin could also participate in hormonal disturbances at the origin of PCOS pathogenesis. Indeed, this adipokine has been identified in the arcuate supraoptic and paraventricular hypothalamic nuclei [62], it has been demonstrated to suppress LH secretion in rats [65], and a negative correlation between plasma apelin and LH levels has repeatedly been shown in humans [66,67]. In our study, FF concentration and GC mRNA expression of apelin was strongly positively correlated with each other and with APJ expression. Further, even if plasma apelin levels largely depend on the dosage method, FF apelin concentration seems to be higher than the plasma one [59]. Thus, follicular apelin may partly derive by GC production and act in a paracrine and/or autocrine manner on GCs themselves. 

### 3.5. Omentin

Omentin, also known as intelectin-1, is a novel adipokine identified from the cDNA library of omental adipose tissue by Yang et al. and predominantly produced by visceral fat depots [68]. Despite some discordant results [29], serum omentin levels have been shown to be inversely related to obesity [69,70] and to increase after weight loss [71]. Contrarily to what was reported in plasma, in our study, we found that FF concentration and GC mRNA expression of omentin were significantly higher in obese women compared to the normal-weight ones and positively correlated with BMI. We also demonstrated that omentin expression in both FF and GCs was significantly higher in women with PCOS compared to controls and ECHO women. These results, already shown by Cloix et al. [18], once again disagree with literature data concerning plasma omentin levels. Indeed, most of the studies investigating omentin expression in PCOS women found lower plasma omentin concentrations [28,29,69,70], as well as decreased omentin mRNA and protein levels in adipose tissue [72]. Several factors have been evoked to explain such results. First, it has been repeatedly reported that serum omentin levels are inversely related with HOMA-IR/fasting insulin [29,69,70,72] and an in vitro study supported the role of hyperinsulinemia in lessening omentin expression in adipose tissue [72]. Hormonal disturbances, and notably hyperandrogenism, have been suggested as another key factor contributing to a decrease of omentin synthesis in PCOS women [29,70]. Indeed, as for some other adipokines, plasma omentin levels are higher in women than in men [73] and negatively correlate with androgens’ levels [29,70]. At last, omentin may also be regulated by inflammation, as its expression is altered in inflammatory states [28] and PCOS is actually considered as a proinflammatory condition [74]. According to these observations, the higher omentin expression that we found in FF and GCs in PCOS women suggests that omentin production at the ovarian level is independent from insulin action and differently regulated in these patients. Furthermore, in our study, FF concentration and GC expression of omentin positively correlated with each other, strongly supporting the hypothesis that follicular omentin is at least partially produced by GCs. Interestingly, Cloix et al. showed that in PCOS women, but not in controls, omentin concentration is doubled in FF compared to in plasma [18]. Whether omentin is implicated in PCOS pathogenesis is, however, still to be demonstrated. In humans, omentin is expressed in reproductive tissues, including the placenta and ovary [70], and it has been shown to enhance, through induction of visfatin expression, GC IGF-1-induced steroidogenesis and IGF-1R signaling [18]. Thus, it has been suggested that omentin and visfatin, modulating insulin sensitivity in GC, could affect ovarian function. Indeed, insulin and IGF-1 act synergistically with FSH to increase GC estrogens’ synthesis and with LH to enhance theca cells’ androgen production [18]. Interestingly, circulating omentin has already been shown to be positively correlated with serum estradiol and negatively correlated with the LH/FSH ratio [29]. Mahde et al. also found higher serum omentin levels in PCOS women with irregular cycles compared to those with regular ones [69], data that agree with our finding of a positive correlation between follicular omentin and cycle duration, further confirming the possible role of this adipokine in ovulatory dysfunction characteristics of PCOS.

### 3.6. Chemerin 

Chemerin, also known as Retinoic Acid Receptor Responder protein 2 (RARRES2), is a small chemotactic protein originally identified as the natural ligand of the G-protein coupled receptor CMKLR1 [75]. Initially known as a proinflammatory cytokine involved in adaptative and innate immunity [76], in 2007, it was discovered as a novel adipokine associated with obesity and metabolic syndrome [77] and shown to promote adipogenesis and adipocyte metabolism [78]. In addition to CMKLR1, two other receptors, GPR1 and CCRL2, were reported to bind chemerin with high affinity, but at present data concerning their functional relevance are poor [75]. In our study, chemerin expression in both FF and GCs was greater in obese women than in the normal-weight ones and strongly positively correlated with BMI. This is consistent with Bozaoglu et al.’s data, showing that chemerin and CMKLR1 were highly expressed in mature adipocytes and upregulated in the adipose tissue of obese animals [77]. Circulating chemerin levels have also been reported to be higher in obese subjects compared to those with a normal BMI and significantly correlated with metabolic syndrome parameters, such as BMI, triglycerides, and fasting serum insulin [23]. Nevertheless, BMI alone does not seem to be a predictive factor for circulating chemerin [31]. Indeed, the existence of a correlation between this adipokine and PCOS has repeatedly been evoked [79]. Our findings showed that both chemerin FF concentration and chemerin and CMKLR1 mRNA levels in GCs were predominant in women with PCOS. The same results have recently been found by Wang et al. in a cohort of non-obese women [21]. Further, serum and ovarian chemerin levels have been shown to be elevated in a dihydrotestosterone (DHT)-induced rat PCOS model [21] and, despite few discordant data [31], the literature widely reports higher chemerin levels in the plasma and adipose tissue of PCOS women [22]. Chemerin has been demonstrated to act as an important negative regulator of ovarian steroidogenesis [80], inhibiting IGF-1-induced secretion of progesterone and estradiol in human GCs [15] and suppressing FSH-induced expression of aromatase and P450scc in cultured rat preantral follicles and GCs [21,81]. Furthermore, in DHT-treated rats, elevated chemerin levels and down-regulated aromatase expression were positively related to increased GC apoptosis [80], suggesting that chemerin may be involved in the antral follicular growth arrest associated to the hyperandrogenic proinflammatory state characterizing PCOS [82]. While keeping in mind that correlations may be merely spurious, without causative significance, it is noteworthy that in our study CMKLR1 expression in GCs was significantly positively correlated with follicle count, cycle duration, and plasma AMH, LH, and estradiol levels. As for apelin and omentin, we found that the follicular concentration of chemerin strongly positively correlated with its mRNA levels in GCs, suggesting that follicular chemerin may be partly produced in the ovary. Indeed, chemerin and CMKLR1 expression has been demonstrated in human and mouse placenta [78], in human, mouse, and rat ovary [21], and more recently in human granulosa and theca cells [15]. In support of this hypothesis, the concentration of this adipokine has been shown to be higher in FF than in plasma [15] and the rise of follicular chemerin in PCOS women has been demonstrated to be independent of changes in its plasma concentration and adiposity, strongly indicating that chemerin is independently regulated at the ovarian level [82]. Interestingly, in our study, CMKLR1 and chemerin expression seemed to be inversely regulated as regards adiposity. Indeed, contrarily to chemerin, CMKLR1 mRNA levels were higher in GCs of normal-weight women and negatively correlated with BMI. Further, chemerin and CMKLR1 levels in GCs negatively correlated with each other, suggesting the possibility of a negative feedback between chemerin and its receptor. Such a regulatory mechanism has already been proposed by Bozaoglu et al. in adipose tissue, but finally, chemerin’s role in the regulation of CMKLR1 expression in adipocytes seems quite limited [77]. As an alternative, these findings could be due to a resistance mechanism to chemerin action at the ovarian level. Indeed, as hyperinsulinemia in the IR states, the high chemerin levels found in obese women could be a compensatory response to the lack of CMKLR1 in GCs. This is, however, a pure hypothesis, since no data is at the moment available about whether and how this chemerin resistance may occur.

### 3.7. ECHO Condition

In our study, we chose to compare women with PCOS and controls to a third group of women presenting at least 12 small antral follicles per ovary without any other criterium necessary for PCOS diagnosis, i.e., hyperandrogenism and/or oligo-anovulation. Besides allowing us to better understand adipokines’ role in PCOS physiopathology, the “ECHO condition” has relevant clinical interest. Indeed, despite some discordant results [83], it has repeatedly been reported to be associated with hyperandrogenism and IR [84,85]. PCOM is also encountered in about 30% of young asymptomatic women [85], but its actual meaning in this condition is currently unknown [86]. There is little evidence to suggest that its sole presence has any significant risk to subsequent health, except for circumstances in which women with PCOM require a gonadotropin treatment, such as during an IVF procedure [86]. Indeed, due to polycystic ovaries’ extreme sensitivity to FSH, an antral follicle count greater than 24 is considered a major risk factor of ovarian hyperstimulation syndrome and nowadays is routinely recommended for the pre-treatment identification of patients at risk [38]. Interestingly, we found that FF concentration and GC mRNA expression of omentin, chemerin, and APJ (for the last two, these observations were limited to the obese group) were significantly lower in ECHO compared to PCOS women, suggesting that these molecules could play a physiopathological role in other main features of PCOS, such as anovulatory infertility. On the contrary, the ECHO group was characterized by higher FF adiponectin levels, possibly reflecting a lower IR compared to the PCOS group or, more intriguingly, a beneficial effect of adiponectin on follicular maturation and subsequent ovulation. Surprisingly, we also observed that, despite no difference in terms of follicle numbers between the two groups, ECHO women presented lower plasma AMH levels than women with PCOS. It has been demonstrated that AMH plays a key role in protecting growing follicles from premature maturation, directly by inhibiting the recruitment of primordial follicles and indirectly by opposing the effects of FSH. For this reason, it is considered an endocrine marker of the number of small antral follicles [87]. Indeed, plasma AMH concentration was reported to be two to three-fold higher in women with PCOS than in normal ovulatory women [88] and in vitro studies demonstrated that GCs of normo-ovulatory and anovulatory PCOS women produce, respectively, 4-fold and 75-fold higher AMH levels compared to controls [89]. Serum AMH levels are therefore considered to reflect the severity of PCOS [90] and notably, the ovulatory disturbances characteristic of this disease [87]. In addition, Homburg et al. reported that AMH levels can be used to differentiate women with PCOS from women with PCOM alone and controls [91], further strengthening our results. As for AMH, plasma LH levels in the ECHO group were intermediate between controls and women with PCOS. Women with PCOM had already been reported to present a hormonal profile, as well as a per-follicle AMH production, intermediate between normal and PCOS women, suggesting that isolated PCOM might represent a PCOS-like phenotype characterized by a mild GC dysfunction, not yet sufficient to affect the ovulatory process [86]. Our findings also agree with literature data showing a tight positive correlation between LH and AMH [86] and support the role of AMH in hormonal alterations at the origin of the ovulatory dysfunction observed in women with PCOS [92].

### 3.8. Limitations and Perspectives 

Although this study provided meaningful information, it has some limitations. Firstly, it was a retrospective and observational study and, as such, it did not permit determination of whether the modifications in adipokines’ concentration were at the origin of the development of polycystic ovaries in PCOS, or rather a consequence or a compensatory response. Secondly, we studied only women requiring IVF and hence under controlled ovarian stimulation, a condition that surely modifies the normal functions of the female reproductive system. Thus, our results might not be generalized to all PCOS and ECHO women. Finally, data about the IR state were not available for most of the women included in the study. This could have given us more information about patients’ hormonal profile and, mostly, helped us to better understand and interpret adipokines’ modifications. Our current understanding of the role of chemerin, omentin, and apelin in PCOS is far from complete and deserves further studies. Notably, it would be of importance to elucidate the molecular mechanisms involved in their effects on GCs and, above all, to study their expression and action on theca cells, which play a key role in the hyperandrogenism characterizing PCOS women.

## 4. Materials and Methods 

### 4.1. Ethics Approval

Study was conducted according to principles set in the Declaration of Helsinki. Informed consent was signed by each participant and study protocol was approved by the Institutional Review Board (Authorization protocol 2016_075, 1 January 2016) Ethic Committee of University Hospital of Tours, France).

### 4.2. Study Population

Biological samples and clinical data of a total of 78 women undergoing an in vitro fertilization (IVF) procedure between 2011 and 2017 at the referral center for reproductive medicine of University Hospital of Tours were analyzed. 

Three groups of patients were created: 23 women suffering from PCOS (PCOS group), 28 women presenting ≥12 follicles per ovary on transvaginal ultrasound without other criteria necessary for diagnosis of PCOS (ECHO group), and 27 women affected by another cause of infertility requiring a MAR procedure (control group). Each group almost equally included normal weight (BMI 18–25 kg/m^2^) and obese (BMI > 30 kg/m^2^) women. A total of 6 groups was then analyzed. Diagnosis of PCOS followed 2003 Rotterdam Consensus Conference Criteria [2]. All women with PCOS presented an oligo/anovulation and follicle count ≥12 per ovary. Clinical and/or biological hyperandrogenism was reported for 6 out of 23 of them. Signs of clinical hyperandrogenism included acne, alopecia, and hirsutism as evaluated by a physician experienced in the field. No woman included in the ECHO and control groups had clinical and/or biological hyperandrogenism and all were characterized by normal ovulatory cycles. They underwent an IVF procedure because of male infertility, tubal sterility, ovarian insufficiency, endometriosis, or a combination of male and female causes. The antral follicle number was assessed in the early follicular phase by transvaginal ultrasound scans of the ovaries performed by experienced sonographers. Blood samples for hormonal evaluations were collected between day 3 and day 5 of the menstrual cycle before the IVF stimulation protocol. Plasma levels of testosterone, oestradiol (E2), luteinizing hormone (LH), and follicle-stimulating hormone (FSH) were measured using an Immulite^®^ 2500 immunoassay analyzer (Siemens, Munich, Germany). However, none of the control and ECHO women included in our study showed clinical and biological hyperandrogenism. Plasma anti-Mullerian hormone (AMH) was determined by Eurofin Biomnis (Lyon, France).

### 4.3. Collection of FF Samples and Isolation of GCs 

GCs were obtained from preovulatory follicles during oocyte retrieval preceding IVF. The controlled ovarian stimulation protocol and IVF procedures employed have already been reported [93]. After isolation of cumulus-oocyte complexes, FF was recovered and centrifuged (400× *g*, 10 min) to separate cell remnants. The supernatant was stored at −80 °C for later analyses. GCs were isolated from erythrocytes with 20 min of centrifugation at 400× *g* on two layers of discontinuous Percoll gradient (40%, 60% in Ham’s F-12 medium; GIBCO-BRL/Life Technologies, Lyon, France). The 40% fraction was collected and treated with hemolytic medium (NH4Cl 10 mM in Tris HCL, pH 7.5; Sigma Aldrich, Saint Quentin-Fallavier, France) to remove the remaining erythrocytes. Following centrifugation, the pellet was washed with fresh medium (Ham’s F-12) and stored at −80 °C for later use.

### 4.4. Adipokines Concentration in FF 

Adiponectin, chemerin, resistin, visfatin, omentin, and apelin concentrations were measured by ELISA in FF samples. ELISA R&D Bio-Techne Ltd. kits (Abingdon, United Kingdom, intra-assay coefficients of variations <6% and inter-assay coefficients of variations ≤8%) were used for all adipokines.

### 4.5. RNA Extraction and Real-Time Quantitative PCR (qPCR)

Total RNA from GCs was extracted with TRIzol reagent according to the manufacturer’s procedure (Sigma Aldrich, Saint Quentin-Fallavier, France). The concentration and the purity of isolated RNA were determined with a NanoDrop spectrophotometer (Peqlab Biotechnologie GmbH, Erlangen, Germany). The integrity of RNA was checked on 1.25% agarose-formaldehyde gels. The cDNA was generated by reverse transcription (RT) of total RNA (1 µg) and real-time quantitative PCR was performed as reported previously [18]. Briefly, total RNA (1 µg) was denatured and reverse transcripted in a 20 µL reaction mixture containing 50 mM Tris-HCL (pH 8.3), 75 mM KCL, 3 mM MgCl_2_, 200 mM of each deoxynucleotide triphosphate, 50 pmol of oligo(dT), 15.5 IU of ribonuclease inhibitor, and 15 IU of Moloney Murine Leukaemia Virus (M-MLV) reverse transcriptase. The mixture was incubated for 1 h at 37 °C. Targeted cDNAs were quantified by real-time PCR using SYBR Green Supermix (Bio-Rad, Marnes la Coquette, France) and 250 nM of specific primers (as mentioned below) in a total volume of 20 µL in a MyiQ Cycle device (Bio-Rad). Samples were tested in duplicate on the same plate and PCR amplification with water instead of cDNA was done systemically as a negative control. After incubation for 2 min at 50 °C and a denaturation step of 10 min at 95 °C, samples were subjected to 40 cycles (30 s at 95 °C, 30 s at 60 °C, 30 s at 72 °C), followed by the acquisition of the melting curve. The levels of mRNA expression were standardized to the geometric mean of three reference genes (GAPDH, beta-actin, and PPIA), which has been reported as an accurate normalization procedure [94]. The relative amounts of gene transcripts (R) were calculated according to the equation: R = (E_gene_^−Ct gene^)/ (geometric mean (E_GAPDH_^−Ct GAPDH^; E_BETA ACTIN_^−Ct BETA ACTIN^; E_PPIA_^−Ct PPIA^)), where E is the primer efficiency and Ct is the cycle threshold.

The primers’ efficiency (E) was performed from serial dilutions of a pool of obtained cDNA and ranged from 1.8 to 2. The specific primer pairs used were: Adiponectin: Fw (forward) 5′-GAAAGGAGATCCAGGTCTTATTG-3′ and Rev (reverse) 5′-TCAGCAAAACCACTATGATGG-3′; AdipoR1: Fw 5′-TTCTTCCTCATGGCTGTGATGT-3′ and Rev 5′-AAGAAGCGCTCAGG-AATTCG-3′; AdipoR2; Fw 5′-CCACCACCTTGCTTCATCTA-3′ and Rev 5′-GATACTGAGGGGTGGCAAAC-3′; visfatin or NAMPT: Fw 5′-CAGCAGAACACAGTACCATA-3′ and Rev 5′-CTCTAAGATAAGGTGGCAGC-3′; omentin: Fw 5′-GGATTTGTTCAGTTCAGGGTATTTAA-3′ and Rev 5′-GCCTCTGGAAAGTATCCTCCT-3′; resistin Fw: 5′-GGACAGGAGCTAATACCCAGAAC-3′ and Rev 5′-GGAAAAGGAGGGGAAATGAA-3′; apelin: Fw 5′-CTCTGGCTCTCCTTGACCG-3′ and Rev 5′-GGCCCATTCCTTGACCCTC-3′; apelin receptor (APJ): Fw 5′-CTATCCTGTTTTCTGAGTGTGAGG-3′ and Rev 5′-CTAAGGGCTGGAGCACTAATTATC-3′; chemerin (rarres2): Fw 5′-CCCAATGGGAGGAAACG-3′ and Rev 5′-CCAGGGAAGTAGAAGCTGTG-3′; CMKLR1: Fw 5′-CCCAATCCATATCACCTATGCC-3′ and Rev 5′-GTCCCGAAAACCCAGTGGTA-3′; CCRL2: Fw 5′-CACATAACTAGGAAGTGGCAGAAC-3′ and Rev 5′-AGCGTAGGCTCTGACCAAAT-3′; GPR1:Fw 5′-CTGTCATTTGGTTCACAGGA-3′ and Rev 5′-AACAACCTGAGGTCCACATC-3′; GADPH: Fw 5′-TGCACCACCAACTGCTTAGC-3′ and Rev 5′GGCATGGACTGTGGTCATGAG-3′; PPIA: Fw 5′-CTGAGCACTGGAGAGAAAGG-3′ and Rev 5′-AGGAATGATCTGGTGGTTAAG-3′; beta Actin; Fw 5′-CTTCTACAATGAGCTGCGTGTG-3′ and Rev 5′- GTGAGGATCTTCATGAGGTAGTCAGTC-3′. PCR products were analyzed on an agarose gel (1.5%) stained with ethidium bromide and the cDNA fragment of interest was confirmed after sequencing by Genewiz (Leipzig, Germany).

### 4.6. Statistical Analyses

Values are reported as mean ± standard deviation (SD). Statistical analyses included two-way ANOVA, followed by Bonferroni post-hoc tests. For each parameter, we examined the effect of BMI and pathological condition, and the interaction between these two parameters. In case of a not-significant BMI effect and no interaction, the data for subsequent analysis were pooled in three groups according to pathological status (controls, PCOS, and ECHO). In all other cases, normal-weight and obese groups were investigated separately. Correlations were analyzed by simple regression analysis and the correlation-Z-test. Neither adjustment for age nor any other factor was made. StatView software (version version 9.3, Distributors: SAS Institute Inc., SAS Campus Drive, Cary NC 27513, USA) was used, with *p* < 0.05 as a threshold statistically significant level.

## 5. Conclusions

In conclusion, we showed in a cohort of women affected by infertility that chemerin and omentin expression in FF and GCs is electively increased in the PCOS group, while ECHO women are characterized by high levels of adiponectin, apelin, and APJ, as well as lower plasma AMH and LH levels. We also demonstrated that the regulation at the ovarian level of these adipokines differs from the systemic one, suggesting that follicular chemerin, omentin, and apelin may be at least partly produced by GCs and act in an autocrine and/or paracrine manner on ovarian follicles’ cells, modulating their functions and, in particular, steroid production. FF concentration of all adipokines varied according to BMI, with resistin, visfatin, chemerin, omentin, and apelin levels higher in obese subjects in contrast with a predominant adiponectin expression in normal-weight women. Our findings thus provide novel insights into the role of chemerin, omentin, and apelin in follicular growth arrest and ovulatory dysfunction characterizing PCOS pathogenesis.

## Figures and Tables

**Table 1:** Study population’s (*n* = 78) clinical parameters, hormonal data, and in vitro fertilization procedure outcomes.

Parameter	Age (y)	BMI (kg/m^2^)	Cycle Duration (d)	FSH (UI/L)	Estradiol (ng/L)	Testosterone (µg/L)
**NW Controls** (*n* = 12)	31.08 ± 3.82	21.77 ± 2.07	28.46 ± 2.15	6.78 ± 3.19	43.33 ± 14.39	0.33 ± 0.20 (*n* = 5)
**NW ECHO** (*n* = 13)	31.69 ± 5.88	20.84 ± 1.86	30.25 ± 2.54	6.40 ± 2.15	42.85 ± 16.01	0.28 ± 0.15 (*n* = 6)
**NW PCOS** (*n* = 13)	29.54 ± 3.36	20.68 ± 1.96	98.54 ± 67.77 ^*^	5.86 ± 1.66	41.55 ± 14.85	0.88 ± 0.93 (*n* = 6)
**Obese Controls** (*n* = 15)	33.80 ± 4.62	33.13 ± 2.29	28.97 ± 2.21	6.71 ± 3.49	43.23 ± 17.24	0.41 ± 0.19 (*n* = 5)
**Obese ECHO** (*n* = 15)	32.73 ± 4.50	31.53 ± 3.33	29.89 ± 1.30	5.68 ± 1.09	35.23 ± 13.43	0.43 ± 0.08 (*n* = 5)
**Obese PCOS** (*n* = 10)	30.10 ± 3.25	33.38 ± 2.12	76.11 ± 61.79 ^#^	4.86 ± 1.64	34.13 ± 6.99	0.49 ± 0.24 (*n* = 7)
**Condition Effect**	*p* = 0.07	*p* = 0.14	*p* < 0.0001	*p* = 0.13	*p* = 0.38	-
**BMI Effect**	*p* = 0.15	*p* < 0.0001	*p* = 0.37	*p* = 0.28	*p* = 0.13	-
**Interaction**	*p* = 0.65	*p* = 0.32	*p* = 0.46	*p* = 0.78	*p* = 0.57	-

Note: BMI = Body Mass Index; FSH = Follicle-Stimulating Hormone; NW = Normal-Weight; * indicated significant difference (*p* < 0.001) between Normal-Weight PCOS women and Normal-Weight ECHO/Control women; # indicated significant difference (*p* < 0.001) between Obese PCOS and Obese ECHO/Control women. The values are expressed as mean ± standard deviation.

**Table 2:** Correlations between follicular fluid concentration of adipokines and clinical parameters, hormonal data, and in vitro fertilization procedure outcomes (*n* = 62).

Parameter	Adiponectin	Chemerin	Resistin	Visfatin	Omentin	Apelin
**BMI (kg/m^2^)**	r = −0.748 ^***^	r = 0.725 ^***^	r = 0.799 ^***^	r = 0.275 ^*^	r = 0.446 ^***^	r = 0.441 ^***^
**Cycle duration (d)**	NS	NS	NS	NS	r = 0.421 ^***^	NS
**Follicles Count (n)**	NS	NS	NS	r = −0.352 ^**^	NS	r = 0.480^* **^
**AMH (ng/mL)**	NS	NS	NS	r = −0.284 ^*^	NS	NS
**Estradiol (ng/L)**	r = −0.300 ^*^	NS	NS	NS	NS	r = −0.284 ^*^
**Oocytes Retrieved (n)**	NS	NS	NS	r = −0.37^**^	NS	r = 0.300^*^
**Embryos (n)**	NS	NS	NS	r = −0.262^*^	NS	r = 0.268^*^

Note: BMI = Body Mass Index; AMH = Anti-Müllerian Hormone. NS = Not Statistically Significant; * *p* < 0.05; ** *p* < 0.01; *** *p* < 0.001.

## References

[1] Teede H., Deeks A., Moran L. (2010). Polycystic ovary syndrome: A complex condition with psychological, reproductive and metabolic manifestations that impacts on health across the lifespan. BMC Med..

[2] Rotterdam ESHRE/ASRM-Sponsored PCOS Consensus Workshop Group (2004). Revised 2003 consensus on diagnostic criteria and long-term health risks related to polycystic ovary syndrome. Fertil. Steril..

[3] Teede H.J., Misso M.L., Costello M.F., Dokras A., Laven J., Moran L., Piltonen T., Norman R.J., International P.N. (2018). Recommendations from the international evidence-based guideline for the assessment and management of polycystic ovary syndrome. Fertil. Steril..

[4] Toulis K.A., Goulis D.G., Farmakiotis D., Georgopoulos N.A., Katsikis I., Tarlatzis B.C., Papadimas I., Panidis D. (2009). Adiponectin levels in women with polycystic ovary syndrome: A systematic review and a meta-analysis. Hum. Reprod. Update.

[5] Benrick A., Chanclon B., Micallef P., Wu Y., Hadi L., Shelton J.M., Stener-Victorin E., Wernstedt Asterholm I. (2017). Adiponectin protects against development of metabolic disturbances in a PCOS mouse model. Proc. Natl. Acad. Sci. USA.

[6] Pasquali R., Pelusi C., Genghini S., Cacciari M., Gambineri A. (2003). Obesity and reproductive disorders in women. Hum. Reprod. Update.

[7] Norman J.E. (2010). The adverse effects of obesity on reproduction. Reproduction.

[8] Pasquali R., Gambineri A., Pagotto U. (2006). The impact of obesity on reproduction in women with polycystic ovary syndrome. BJOG.

[9] Bluher M. (2009). Adipose tissue dysfunction in obesity. Exp. Clin. Endocrinol. Diabetes.

[10] Ohashi K., Shibata R., Murohara T., Ouchi N. (2014). Role of anti-inflammatory adipokines in obesity-related diseases. Trends. Endocrinol. Metab..

[11] Vazquez M.J., Romero-Ruiz A., Tena-Sempere M. (2015). Roles of leptin in reproduction, pregnancy and polycystic ovary syndrome: Consensus knowledge and recent developments. Metabolism.

[12] Landry D., Cloutier F., Martin L.J. (2013). Implications of leptin in neuroendocrine regulation of male reproduction. Reprod. Biol..

[13] Dupont J., Pollet-Villard X., Reverchon M., Mellouk N., Levy R. (2015). Adipokines in human reproduction. Horm. Mol. Biol. Clin. Investig..

[14] Chabrolle C., Tosca L., Rame C., Lecomte P., Royere D., Dupont J. (2009). Adiponectin increases insulin-like growth factor I-induced progesterone and estradiol secretion in human granulosa cells. Fertil. Steril..

[15] Reverchon M., Cornuau M., Rame C., Guerif F., Royere D., Dupont J. (2012). Chemerin inhibits IGF-1-induced progesterone and estradiol secretion in human granulosa cells. Hum. Reprod..

[16] Reverchon M., Cornuau M., Rame C., Guerif F., Royere D., Dupont J. (2013). Resistin decreases insulin-like growth factor I-induced steroid production and insulin-like growth factor I receptor signaling in human granulosa cells. Fertil. Steril..

[17] Reverchon M., Cornuau M., Cloix L., Rame C., Guerif F., Royere D., Dupont J. (2013). Visfatin is expressed in human granulosa cells: Regulation by metformin through AMPK/SIRT1 pathways and its role in steroidogenesis. Mol. Hum. Reprod..

[18] Cloix L., Reverchon M., Cornuau M., Froment P., Rame C., Costa C., Froment G., Lecomte P., Chen W., Royere D. (2014). Expression and regulation of INTELECTIN1 in human granulosa-lutein cells: Role in IGF-1-induced steroidogenesis through NAMPT. Biol. Reprod..

[19] Roche J., Rame C., Reverchon M., Mellouk N., Cornuau M., Guerif F., Froment P., Dupont J. (2016). Apelin (APLN) and Apelin Receptor (APLNR) in Human Ovary: Expression, Signaling, and Regulation of Steroidogenesis in Primary Human Luteinized Granulosa Cells. Biol. Reprod..

[20] Tang M., Huang C., Wang Y.F., Ren P.G., Chen L., Xiao T.X., Wang B.B., Pan Y.F., Tsang B.K., Zabel B.A. (2016). CMKLR1 deficiency maintains ovarian steroid production in mice treated chronically with dihydrotestosterone. Sci. Rep..

[21] Wang Q., Kim J.Y., Xue K., Liu J.Y., Leader A., Tsang B.K. (2012). Chemerin, a novel regulator of follicular steroidogenesis and its potential involvement in polycystic ovarian syndrome. Endocrinology.

[22] Tan B.K., Chen J., Farhatullah S., Adya R., Kaur J., Heutling D., Lewandowski K.C., O’Hare J.P., Lehnert H., Randeva H.S. (2009). Insulin and metformin regulate circulating and adipose tissue chemerin. Diabetes.

[23] Bozaoglu K., Segal D., Shields K.A., Cummings N., Curran J.E., Comuzzie A.G., Mahaney M.C., Rainwater D.L., VandeBerg J.L., MacCluer J.W. (2009). Chemerin is associated with metabolic syndrome phenotypes in a Mexican-American population. J. Clin. Endocrinol. Metab..

[24] Munir I., Yen H.W., Baruth T., Tarkowski R., Azziz R., Magoffin D.A., Jakimiuk A.J. (2005). Resistin stimulation of 17alpha-hydroxylase activity in ovarian theca cells in vitro: Relevance to polycystic ovary syndrome. J. Clin. Endocrinol. Metab..

[25] Baldani D.P., Skrgatic L., Kasum M., Zlopasa G., Kralik Oguic S., Herman M. (2019). Altered leptin, adiponectin, resistin and ghrelin secretion may represent an intrinsic polycystic ovary syndrome abnormality. Gynecol. Endocrinol..

[26] Chan T.F., Chen Y.L., Chen H.H., Lee C.H., Jong S.B., Tsai E.M. (2007). Increased plasma visfatin concentrations in women with polycystic ovary syndrome. Fertil. Steril..

[27] Panidis D., Farmakiotis D., Rousso D., Katsikis I., Delkos D., Piouka A., Gerou S., Diamanti-Kandarakis E. (2008). Plasma visfatin levels in normal weight women with polycystic ovary syndrome. Eur. J. Intern. Med..

[28] Tan B.K., Adya R., Farhatullah S., Chen J., Lehnert H., Randeva H.S. (2010). Metformin treatment may increase omentin-1 levels in women with polycystic ovary syndrome. Diabetes.

[29] Orlik B., Madej P., Owczarek A., Skalba P., Chudek J., Olszanecka-Glinianowicz M. (2014). Plasma omentin and adiponectin levels as markers of adipose tissue dysfunction in normal weight and obese women with polycystic ovary syndrome. Clin. Endocrinol. (Oxf).

[30] Barbe A., Bongrani A., Mellouk N., Estienne A., Kurowska P., Grandhaye J., Elfassy Y., Levy R., Rak A., Froment P. (2019). Mechanisms of Adiponectin Action in Fertility: An. Overview from Gametogenesis to Gestation in Humans and Animal Models in Normal and Pathological Conditions. Int. J. Mol. Sci..

[31] Guvenc Y., Var A., Goker A., Kuscu N.K. (2016). Assessment of serum chemerin, vaspin and omentin-1 levels in patients with polycystic ovary syndrome. J. Int. Med. Res..

[32] Farshchian F., Ramezani Tehrani F., Amirrasouli H., Rahimi Pour H., Hedayati M., Kazerouni F., Soltani A. (2014). Visfatin and resistin serum levels in normal-weight and obese women with polycystic ovary syndrome. Int. J. Endocrinol. Metab..

[33] Akbarzadeh S., Ghasemi S., Kalantarhormozi M., Nabipour I., Abbasi F., Aminfar A., Jaffari S.M., Motamed N., Movahed A., Mirzaei M. (2012). Relationship among plasma adipokines, insulin and androgens level as well as biochemical glycemic and lipidemic markers with incidence of PCOS in women with normal BMI. Gynecol. Endocrinol..

[34] Kim J.J., Choi Y.M., Hong M.A., Kim M.J., Chae S.J., Kim S.M., Hwang K.R., Yoon S.H., Ku S.Y., Suh C.S. (2018). Serum visfatin levels in non-obese women with polycystic ovary syndrome and matched controls. Obstet. Gynecol. Sci..

[35] Tao T., Xu B., Liu W. (2013). Ovarian HMW adiponectin is associated with folliculogenesis in women with polycystic ovary syndrome. Reprod. Biol. Endocrinol..

[36] Tsouma I., Kouskouni E., Demeridou S., Boutsikou M., Hassiakos D., Chasiakou A., Hassiakou S., Baka S. (2014). Correlation of visfatin levels and lipoprotein lipid profiles in women with polycystic ovary syndrome undergoing ovarian stimulation. Gynecol. Endocrinol..

[37] Seow K.M., Juan C.C., Hsu Y.P., Ho L.T., Wang Y.Y., Hwang J.L. (2005). Serum and follicular resistin levels in women with polycystic ovarian syndrome during IVF-stimulated cycles. Hum. Reprod..

[38] Practice Committee of the American Society for Reproductive Medicine (2016). Prevention and treatment of moderate and severe ovarian hyperstimulation syndrome: A guideline. Fertil. Steril..

[39] Li S., Huang X., Zhong H., Peng Q., Chen S., Xie Y., Qin X., Qin A. (2014). Low circulating adiponectin levels in women with polycystic ovary syndrome: An updated meta-analysis. Tumour. Biol..

[40] Artimani T., Saidijam M., Aflatoonian R., Ashrafi M., Amiri I., Yavangi M., SoleimaniAsl S., Shabab N., Karimi J., Mehdizadeh M. (2016). Downregulation of adiponectin system in granulosa cells and low levels of HMW adiponectin in PCOS. J. Assist. Reprod. Genet..

[41] O’Connor A., Phelan N., Tun T.K., Boran G., Gibney J., Roche H.M. (2010). High-molecular-weight adiponectin is selectively reduced in women with polycystic ovary syndrome independent of body mass index and severity of insulin resistance. J. Clin. Endocrinol. Metab..

[42] Campos D.B., Palin M.F., Bordignon V., Murphy B.D. (2008). The ‘beneficial’ adipokines in reproduction and fertility. Intj. Obes. (Lond.).

[43] Patel L., Buckels A.C., Kinghorn I.J., Murdock P.R., Holbrook J.D., Plumpton C., Macphee C.H., Smith S.A. (2003). Resistin is expressed in human macrophages and directly regulated by PPAR gamma activators. Biochem. Biophys. Res. Commun..

[44] Fain J.N., Cheema P.S., Bahouth S.W., Lloyd Hiler M. (2003). Resistin release by human adipose tissue explants in primary culture. Biochem. Biophys. Res. Commun..

[45] Panidis D., Koliakos G., Kourtis A., Farmakiotis D., Mouslech T., Rousso D. (2004). Serum resistin levels in women with polycystic ovary syndrome. Fertil. Steril..

[46] Lee J.H., Chan J.L., Yiannakouris N., Kontogianni M., Estrada E., Seip R., Orlova C., Mantzoros C.S. (2003). Circulating resistin levels are not associated with obesity or insulin resistance in humans and are not regulated by fasting or leptin administration: Cross-sectional and interventional studies in normal, insulin-resistant, and diabetic subjects. J. Clin. Endocrinol. Metab..

[47] Seow K.M., Juan C.C., Hsu Y.P., Hwang J.L., Huang L.W., Ho L.T. (2007). Amelioration of insulin resistance in women with PCOS via reduced insulin receptor substrate-1 Ser312 phosphorylation following laparoscopic ovarian electrocautery. Hum. Reprod..

[48] Seow K.M., Juan C.C., Wu L.Y., Hsu Y.P., Yang W.M., Tsai Y.L., Hwang J.L., Ho L.T. (2004). Serum and adipocyte resistin in polycystic ovary syndrome with insulin resistance. Hum. Reprod..

[49] Samal B., Sun Y., Stearns G., Xie C., Suggs S., McNiece I. (1994). Cloning and characterization of the cDNA encoding a novel human pre-B-cell colony-enhancing factor. Mol. Cell. Biol..

[50] Fukuhara A., Matsuda M., Nishizawa M., Segawa K., Tanaka M., Kishimoto K., Matsuki Y., Murakami M., Ichisaka T., Murakami H. (2005). Visfatin: A protein secreted by visceral fat that mimics the effects of insulin. Science.

[51] Berndt J., Kloting N., Kralisch S., Kovacs P., Fasshauer M., Schon M.R., Stumvoll M., Bluher M. (2005). Plasma visfatin concentrations and fat depot-specific mRNA expression in humans. Diabetes.

[52] Chen M.P., Chung F.M., Chang D.M., Tsai J.C., Huang H.F., Shin S.J., Lee Y.J. (2006). Elevated plasma level of visfatin/pre-B cell colony-enhancing factor in patients with type 2 diabetes mellitus. J. Clin. Endocrinol. Metab..

[53] Shen C.J., Tsai E.M., Lee J.N., Chen Y.L., Lee C.H., Chan T.F. (2010). The concentrations of visfatin in the follicular fluids of women undergoing controlled ovarian stimulation are correlated to the number of oocytes retrieved. Fertil. Steril..

[54] Plati E., Kouskouni E., Malamitsi-Puchner A., Boutsikou M., Kaparos G., Baka S. (2010). Visfatin and leptin levels in women with polycystic ovaries undergoing ovarian stimulation. Fertil. Steril..

[55] Tan B.K., Chen J., Digby J.E., Keay S.D., Kennedy C.R., Randeva H.S. (2006). Increased visfatin messenger ribonucleic acid and protein levels in adipose tissue and adipocytes in women with polycystic ovary syndrome: Parallel increase in plasma visfatin. J. Clin. Endocrinol. Metab..

[56] Choi K.H., Joo B.S., Sun S.T., Park M.J., Son J.B., Joo J.K., Lee K.S. (2012). Administration of visfatin during superovulation improves developmental competency of oocytes and fertility potential in aged female mice. Fertil. Steril..

[57] Tatemoto K., Hosoya M., Habata Y., Fujii R., Kakegawa T., Zou M.X., Kawamata Y., Fukusumi S., Hinuma S., Kitada C. (1998). Isolation and characterization of a novel endogenous peptide ligand for the human APJ receptor. Biochem. Biophys. Res. Commun..

[58] Boucher J., Masri B., Daviaud D., Gesta S., Guigne C., Mazzucotelli A., Castan-Laurell I., Tack I., Knibiehler B., Carpene C. (2005). Apelin, a newly identified adipokine up-regulated by insulin and obesity. Endocrinology.

[59] Chang Y.H., Chang D.M., Lin K.C., Shin S.J., Lee Y.J. (2011). Visfatin in overweight/obesity, type 2 diabetes mellitus, insulin resistance, metabolic syndrome and cardiovascular diseases: A meta-analysis and systemic review. Diabetes Metab. Res. Rev..

[60] Rak A., Drwal E., Rame C., Knapczyk-Stwora K., Slomczynska M., Dupont J., Gregoraszczuk E.L. (2017). Expression of apelin and apelin receptor (APJ) in porcine ovarian follicles and in vitro effect of apelin on steroidogenesis and proliferation through APJ activation and different signaling pathways. Theriogenology.

[61] Shirasuna K., Shimizu T., Sayama K., Asahi T., Sasaki M., Berisha B., Schams D., Miyamoto A. (2008). Expression and localization of apelin and its receptor APJ in the bovine corpus luteum during the estrous cycle and prostaglandin F2alpha-induced luteolysis. Reproduction.

[62] Roche J., Rame C., Reverchon M., Mellouk N., Rak A., Froment P., Dupont J. (2017). Apelin (APLN) regulates progesterone secretion and oocyte maturation in bovine ovarian cells. Reproduction.

[63] Shimizu T., Kosaka N., Murayama C., Tetsuka M., Miyamoto A. (2009). Apelin and APJ receptor expression in granulosa and theca cells during different stages of follicular development in the bovine ovary: Involvement of apoptosis and hormonal regulation. Anim. Reprod. Sci..

[64] Franks S., Stark J., Hardy K. (2008). Follicle dynamics and anovulation in polycystic ovary syndrome. Hum. Reprod. Update.

[65] Taheri S., Murphy K., Cohen M., Sujkovic E., Kennedy A., Dhillo W., Dakin C., Sajedi A., Ghatei M., Bloom S. (2002). The effects of centrally administered apelin-13 on food intake, water intake and pituitary hormone release in rats. Biochem. Biophys. Res. Commun..

[66] Altinkaya S.O., Nergiz S., Kucuk M., Yuksel H. (2014). Apelin levels in relation with hormonal and metabolic profile in patients with polycystic ovary syndrome. Eurj. Obstet. Gynecol. Reprod. Biol..

[67] Olszanecka-Glinianowicz M., Madej P., Nylec M., Owczarek A., Szanecki W., Skalba P., Chudek J. (2013). Circulating apelin level in relation to nutritional status in polycystic ovary syndrome and its association with metabolic and hormonal disturbances. Clin. Endocrinol. (Oxf).

[68] Yang R.Z., Lee M.J., Hu H., Pray J., Wu H.B., Hansen B.C., Shuldiner A.R., Fried S.K., McLenithan J.C., Gong D.W. (2006). Identification of omentin as a novel depot-specific adipokine in human adipose tissue: Possible role in modulating insulin action. Am. J. Physiol. Endocrinol. Metab..

[69] Mahde A., Shaker M., Al-Mashhadani Z. (2009). Study of Omentin1 and Other Adipokines and Hormones in PCOS Patients. Oman. Med. J..

[70] Choi J.H., Rhee E.J., Kim K.H., Woo H.Y., Lee W.Y., Sung K.C. (2011). Plasma omentin-1 levels are reduced in non-obese women with normal glucose tolerance and polycystic ovary syndrome. Eur. J. Endocrinol..

[71] Moreno-Navarrete J.M., Catalan V., Ortega F., Gomez-Ambrosi J., Ricart W., Fruhbeck G., Fernandez-Real J.M. (2010). Circulating omentin concentration increases after weight loss. Nutr. Metab. (Lond).

[72] Tan B.K., Pua S., Syed F., Lewandowski K.C., O’Hare J.P., Randeva H.S. (2008). Decreased plasma omentin-1 levels in Type 1 diabetes mellitus. Diabet. Med..

[73] de Souza Batista C.M., Yang R.Z., Lee M.J., Glynn N.M., Yu D.Z., Pray J., Ndubuizu K., Patil S., Schwartz A., Kligman M. (2007). Omentin plasma levels and gene expression are decreased in obesity. Diabetes.

[74] Spritzer P.M., Lecke S.B., Satler F., Morsch D.M. (2015). Adipose tissue dysfunction, adipokines, and low-grade chronic inflammation in polycystic ovary syndrome. Reproduction.

[75] De Henau O., Degroot G.N., Imbault V., Robert V., De Poorter C., McHeik S., Gales C., Parmentier M., Springael J.Y. (2016). Signaling Properties of Chemerin Receptors CMKLR1, GPR1 and CCRL2. PLoS ONE.

[76] Wittamer V., Franssen J.D., Vulcano M., Mirjolet J.F., Le Poul E., Migeotte I., Brezillon S., Tyldesley R., Blanpain C., Detheux M. (2003). Specific recruitment of antigen-presenting cells by chemerin, a novel processed ligand from human inflammatory fluids. J. Exp. Med..

[77] Bozaoglu K., Bolton K., McMillan J., Zimmet P., Jowett J., Collier G., Walder K., Segal D. (2007). Chemerin is a novel adipokine associated with obesity and metabolic syndrome. Endocrinology.

[78] Goralski K.B., McCarthy T.C., Hanniman E.A., Zabel B.A., Butcher E.C., Parlee S.D., Muruganandan S., Sinal C.J. (2007). Chemerin, a novel adipokine that regulates adipogenesis and adipocyte metabolism. J. Biol. Chem..

[79] Kort D.H., Kostolias A., Sullivan C., Lobo R.A. (2015). Chemerin as a marker of body fat and insulin resistance in women with polycystic ovary syndrome. Gynecol. Endocrinol..

[80] Tsatsanis C., Dermitzaki E., Avgoustinaki P., Malliaraki N., Mytaras V., Margioris A.N. (2015). The impact of adipose tissue-derived factors on the hypothalamic-pituitary-gonadal (HPG) axis. Horm. (Athens).

[81] Kim J.Y., Xue K., Cao M., Wang Q., Liu J.Y., Leader A., Han J.Y., Tsang B.K. (2013). Chemerin suppresses ovarian follicular development and its potential involvement in follicular arrest in rats treated chronically with dihydrotestosterone. Endocrinology.

[82] Lima P.D.A., Nivet A.L., Wang Q., Chen Y.A., Leader A., Cheung A., Tzeng C.R., Tsang B.K. (2018). Polycystic ovary syndrome: Possible involvement of androgen-induced, chemerin-mediated ovarian recruitment of monocytes/macrophages. Biol. Reprod..

[83] Dunaif A. (1997). Insulin resistance and the polycystic ovary syndrome: Mechanism and implications for pathogenesis. Endocr. Rev..

[84] Panidis D., Tziomalos K., Misichronis G., Papadakis E., Betsas G., Katsikis I., Macut D. (2012). Insulin resistance and endocrine characteristics of the different phenotypes of polycystic ovary syndrome: A prospective study. Hum. Reprod..

[85] Dewailly D., Pigny P., Soudan B., Catteau-Jonard S., Decanter C., Poncelet E., Duhamel A. (2010). Reconciling the definitions of polycystic ovary syndrome: The ovarian follicle number and serum anti-Mullerian hormone concentrations aggregate with the markers of hyperandrogenism. J. Clin. Endocrinol. Metab..

[86] Dewailly D., Lujan M.E., Carmina E., Cedars M.I., Laven J., Norman R.J., Escobar-Morreale H.F. (2014). Definition and significance of polycystic ovarian morphology: A task force report from the Androgen Excess and Polycystic Ovary Syndrome Society. Hum. Reprod. Update.

[87] Pigny P., Merlen E., Robert Y., Cortet-Rudelli C., Decanter C., Jonard S., Dewailly D. (2003). Elevated serum level of anti-mullerian hormone in patients with polycystic ovary syndrome: Relationship to the ovarian follicle excess and to the follicular arrest. J. Clin. Endocrinol. Metab..

[88] Teede H., Misso M., Tassone E.C., Dewailly D., Ng E.H., Azziz R., Norman R.J., Andersen M., Franks S., Hoeger K. (2019). Anti-Mullerian Hormone in PCOS: A Review Informing International Guidelines. Trends Endocrinol. Metab..

[89] Victoria M., Labrosse J., Krief F., Cedrin-Durnerin I., Comtet M., Grynberg M. (2019). Anti Mullerian Hormone: More than a biomarker of female reproductive function. J. Gynecol. Obstet. Hum. Reprod..

[90] Pierre A., Peigne M., Grynberg M., Arouche N., Taieb J., Hesters L., Gonzales J., Picard J.Y., Dewailly D., Fanchin R. (2013). Loss of LH-induced down-regulation of anti-Mullerian hormone receptor expression may contribute to anovulation in women with polycystic ovary syndrome. Hum. Reprod..

[91] Homburg R., Crawford G. (2014). The role of AMH in anovulation associated with PCOS: A hypothesis. Hum. Reprod..

[92] Cimino I., Casoni F., Liu X., Messina A., Parkash J., Jamin S.P., Catteau-Jonard S., Collier F., Baroncini M., Dewailly D. (2016). Novel role for anti-Mullerian hormone in the regulation of GnRH neuron excitability and hormone secretion. Nat. Commun..

[93] Guerif F., Bidault R., Gasnier O., Couet M.L., Gervereau O., Lansac J., Royere D. (2004). Efficacy of blastocyst transfer after implantation failure. Reprod. Biomed. Online.

[94] Vandesompele J., De Preter K., Pattyn F., Poppe B., Van Roy N., De Paepe A., Speleman F. (2002). Accurate normalization of real-time quantitative RT-PCR data by geometric averaging of multiple internal control genes. Genome Biol..

